# Over-Intake of Plant-Derived Antioxidants, H_2_S Generation, and Reductive Stress. Comment on Manzano-Pech et al. The Chronic Elevated Consumption of *Hibiscus sabdariffa* Linnaeus Results in Kidney Damage Associated with Excess H_2_S. *Int. J. Mol. Sci.* 2026, *27*, 2190

**DOI:** 10.3390/ijms27104285

**Published:** 2026-05-12

**Authors:** Johnny Thai Nguyen, Phuong Phoebe Pham, Liang-Jun Yan

**Affiliations:** Department of Pharmaceutical Sciences, UNT System College of Pharmacy, University of North Texas Health Science Center, Fort Worth, TX 76107, USA; johnnynguyen9@my.unthsc.edu (J.T.N.); phoebepham@my.unthsc.edu (P.P.P.)

Reductive stress is the opposite of oxidative stress but can transition to oxidative stress [1,2,3,4,5,6,7]. The past few decades have witnessed an increasing interest in exploring the mechanisms of reductive stress and its association with aging and metabolic diseases [3,8,9,10,11]. It is established that reductive stress is often caused by a redox imbalance of redox couples such as NAD^+^/NADH, NADP^+^/NADPH, and GSH/GSSG [7,12,13,14]. Perturbation of these redox couples can be attributed to a variety of factors, such as accumulation of NAD(P)H or GSH, impairment of electron transport chain, increased synthesis of antioxidant enzymes, and decreased production of basal level reactive oxygen species that are required for optimal redox signaling under physiological conditions [6,15,16,17].

In a recent study published in this journal, Manzano-Pech et al. demonstrated that H_2_S is another player in reductive stress [18]. The authors fed rats for 1 month with 6% *Hibiscus sabdariffa* Linnaeus (HSL), which is a rich source of antioxidants such as polyphenols and anthocyanins. HSL also provides redox-sensitive amino acids such as serine, cysteine, and methionine. These amino acids can lead to overexpression of cystathionine-β-synthase and cystathionine-γ-lyase, which further drive H_2_S production [18]. The authors further demonstrated that H_2_S was associated with an elevation of cellular reducing power, including NADH, NADPH, GSH, and Nrf2 activation, thereby creating a reductive stress condition that causes kidney damage (Figure 1).

This study, as a continuation of prior work by the same group [19], indicates that while low HSL consumption is beneficial under a variety of pathophysiological conditions, high HSL consumption under healthy conditions is detrimental and this deleterious effect could be reversed if the high consumption of HSL was discontinued. The authors demonstrate that the reductive stress mechanism was due to excess H_2_S that is associated with electrons accumulating at complex I, leading to NADH accumulation and further promoting reductive stress [20,21,22]. This study also provides a useful platform whereby chronic over consumption of HSL can serve as a reductive stress animal model, which should be invaluable for exploring the mechanisms of reductive stress and therapeutic strategies designed to counteract reductive stress-induced disorders. Nevertheless, the study is not without limitations. As indicated by the authors, a dose–response threshold with respect to the transition from eustress to reductive stress is yet to be established. Moreover, potential redox signaling effects of this reductive stress on NAD^+^-dependent enzymes [23,24,25,26] such as sirtuins, CD38, and poly ADP ribosylase also remain to be investigated. Additionally, protein targets impaired by this reductive stress that lead to loss of kidney function also remain to be identified. It should also be pointed out that while the high dose (6% HSL) is toxic in animal studies, whether this high dose would impose toxicity or reductive stress in humans is yet to be assessed.

## Figures and Tables

**Figure 1:**
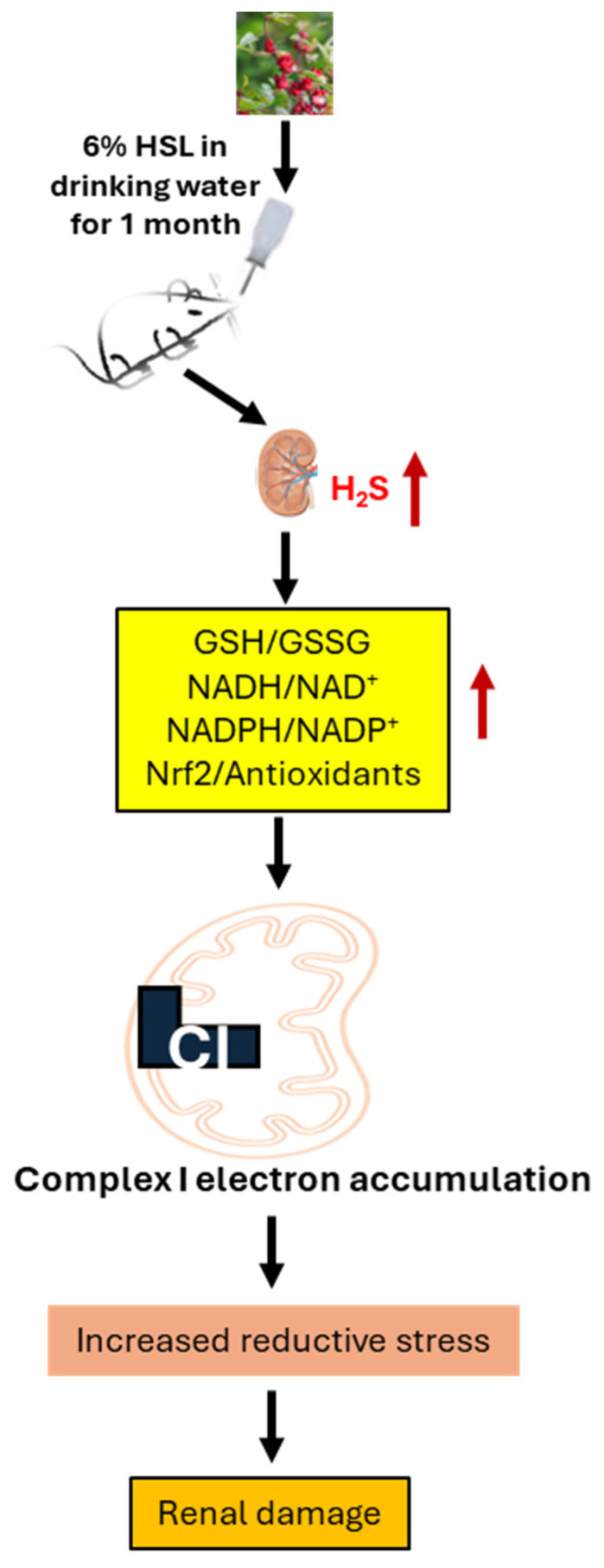
Chronic over-consumption of HSL by rats elevates renal H2S production which increases cellular antioxidant power and causes reductive stress that leads to renal damage. CI: Complex I.
